# The effectiveness of weight loss programs for low back pain: a systematic review

**DOI:** 10.1186/s12891-022-05391-w

**Published:** 2022-05-23

**Authors:** Lu Hsi Chen, Kirsten Weber, Saba Mehrabkhani, Sarmina Baskaran, Thomas Abbass, Luciana Gazzi Macedo

**Affiliations:** School of Rehabilitation Sciences, Faculty of Health Sciences, Institute of Applied Health Sciences, McMaster University, 1400 Main St. W. Hamilton, Hamilton, ON L8S 1C7 Canada

**Keywords:** Low back pain, Weight loss, Systematic review

## Abstract

**Background:**

Low back pain has been associated with obesity or with being overweight. However, there are no high-quality systematic reviews that have been conducted on the effect of all types of weight loss programs focused on individuals with low back pain. Therefore, the present systematic review aims to evaluate the effectiveness of weight loss programs in reducing back pain and disability or increasing quality of life for individuals experiencing low back pain.

**Materials and methods:**

Searches for relevant studies were conducted on CINAHL, Web of Science, Ovid Medline, Ovid Embase and AMED. Studies were included if they were randomized controlled trials, non-randomized studies of intervention or quasi-experimental designs evaluating a weight loss program for persons with low back pain aimed at decreasing back pain and disability. The Effective Public Health Practice Project (EPHPP) Quality Assessment Tool was used to evaluate individual studies and GRADE was used to summarize the quality of the evidence. The review was prospectively registered; PROSPERO#: CRD42020196099.

**Results:**

Eleven studies (*n* = 689 participants) including one randomized controlled trial, two non-randomized studies of intervention and eight single-arm studies were included (seven of which evaluated bariatric surgery). There was low-quality evidence that a lifestyle intervention was no better than waitlist for improving back pain and very low-quality evidence from single-arm studies that back pain improved from baseline after bariatric surgery. Most studies included were of poor quality, primarily due to selection bias, uncontrolled confounders, and lack of blinding, limiting the quality of evidence.

**Conclusion:**

There is very low-quality evidence that weight loss programs may improve back pain, disability, and quality of life in patients with LBP, although adherence and maintenance are potential barriers to implementation.

**Supplementary Information:**

The online version contains supplementary material available at 10.1186/s12891-022-05391-w.

## Introduction

According to the World Health Organization, low back pain (LBP) has reached epidemic proportions, with 80% of people reporting LBP at some time in their life [1]. About one in four persons with LPB are expected to seek care within six months, resulting in considerable social and economic burden [2]. Persons who suffer from either acute or chronic LBP usually have high levels of disability, decreased function and participation, and poor quality of life [3–6]. Consistent guidelines for acute LBP feature early and gradual advice to stay active and avoid prescribing bed rest, while common guidelines for the management of chronic LBP includes supervised exercises, cognitive behavioural therapy, and self-management strategies [6]. In addition to the emphasis on exercise, recent studies suggest that lifestyle modifications should be integrated into LBP management programs [7–9].

There is a growing number of studies suggesting an association between being overweight/obese and having LBP [7, 8, 10–12]. Multiple studies have found that after controlling for potential confounders (e.g., age, sex), the prevalence of LBP is significantly increased in the presence of a high body mass index (BMI) [10, 12, 13]. In addition, a systematic review reported that 32% of 65 epidemiological studies identified a statistically significant positive association between body weight and LBP [14]. One proposed mechanism of association between LBP and weight is that high BMI leads to additional mechanical load on the spine, predisposing individuals to spinal overload [8, 10, 12, 15]. Similarly, there is evidence of a relationship between obesity, systemic inflammation, and LBP, with pro-inflammatory pathways amplified in obesity due to the presence of increased cytokines in adipose tissue [13]. Regardless of the potential pathway through which obesity could be associated with LBP, the findings implicate the vital role that a weight loss program could play in the management of LBP. Ultimately, participating in a weight loss program could translate into a lifestyle change that could not only decrease LBP but also create lifelong benefits in one’s overall health.

Previously, a review of the effects of bariatric surgery on spine pain and upper and lower extremity pain concluded that most of the existing evidence has shown favorable improvements in back pain symptoms after bariatric procedures [16]. However, no systematic literature review is currently available on the effect of all types of weight-loss programs for individuals with LBP. Thus, the present systematic review aims to determine whether a weight loss program is effective in decreasing back pain and disability and/or increasing quality of life in patients with LBP. Given that there are no high-quality systematic reviews that have been conducted on the effect of all types of weight loss programs, we conducted a systematic review of randomized controlled trials (RCTs), non-randomized studies of intervention (NRSI) and quasi-experimental designs such as single-arm studies.

## Materials and methods

This systematic review was reported following the PRISMA guidelines [17] and conducted following the Cochrane Handbook review methods [18].

### Search methods for identification of studies

An electronic search was conducted on CINAHL (1981 to June 2020), Web of Science (1900 to June 2020), Ovid Medline (1946 to June 2020), Ovid Embase (1974 to June 2020), and AMED (1985 to June 2020) to identify relevant articles. The search was not restricted to any specific language or year of publication. Key terms for weight loss and LBP were used and a search strategy was constructed in consultation with an experienced university librarian (Appendix 1). Citation tracking of the included studies was performed using Web of Science (Thomson Reuters). A manual search of the reference lists of previous reviews and eligible trials was also conducted.

### Inclusion Criteria

*Types of participants:* Studies with adults (18 years or older), who are overweight or obese (BMI > 25 kg/m^2^), with LBP, with or without leg pain, of any duration (acute: 0–6 weeks, subacute: 6 -12 weeks and chronic: > 12 weeks) were included. Trials evaluating non-specific LBP, as well as specific conditions, such as radiculopathy or spinal stenosis were included. Trials involving a mixed population where some participants did not have back pain at baseline were included if we could identify data for the subgroup of patients that did have back pain at baseline.

*Types of interventions/comparators:* Studies were included if they evaluated a weight loss program (e.g., physical activity, dietetic treatment) or weight loss treatment (e.g., surgical intervention). If a comparator group existed, the study was included if the comparator group received no treatment, a placebo, or another active treatment (e.g., healthy lifestyle education vs no education).

*Types of outcome measures*: Trials were included if one of the following outcome measures had been reported: presence of LBP, LBP intensity, disability, or quality of life. For studies with a mixed population of participants with and without back pain, when average pain was presented for the whole population rather than the subgroup of back pain patients, the paper was excluded.

### Types of studies

Articles were eligible for inclusion if they were RCTs, NRSIs or quasi-experimental designs such as single-arm studies. Case studies, retrospective chart analysis, gray literature studies (e.g., abstracts, conferences, commentaries, editorials), systematic reviews, and psychometric studies were not eligible for inclusion.

### Data extraction and risk of bias assessment

All authors were involved in screening. Screening for all levels was conducted in duplicate by two review authors who screened all search results (titles and abstracts) for potentially eligible studies and screened full texts for eligibility. Data extraction and assessment of risk of bias was checked by a second author. A third independent reviewer resolved disagreements when necessary. Given the inclusion of multiple study designs, the Effective Public Health Practice Project (EPHPP) Quality Assessment Tool for Quantitative Studies was used to evaluate risk of bias of all studies [19]. Although the Cochrane Handbook suggest the use of different scales to assess the risk of bias of different study designs, given that there was only one RCT in this review, we chose to use the EPHPP tool for all included studies. This quality assessment tool scores six components individually (selection bias, study design, confounders, blinding, data collection method, and withdrawals and dropouts) as either strong, moderate, or weak. A global rating is provided as strong if no individual component is rated as weak, moderate if one of the six components is rated as weak and finally, weak if two or more components are rated as weak. The rating was done based on the EPHPP Quality Assessment Tool Dictionary [19].

Data was extracted from each included study using a standardized extraction form. Mean scores, standard deviations and sample sizes were extracted from the studies when continuous outcomes were reported. Number of events and sample sizes were extracted when dichotomous outcomes were reported. When these results were not presented in the studies, a fixed effects model was used to calculate within or between group differences when possible, using the PEDro Excel sheet calculator [20]. Information about characteristics of participants, treatments provided, co-interventions, duration of the treatment and outcome measures were also extracted from the studies. Given the heterogeneity of the included studies, results were summarized qualitatively using tables.

### Quality of evidence assessment

We assessed the overall quality of evidence using the Grading of Recommendations Assessment, Development and Evaluation (GRADE) approach, as recommended in the *Cochrane Handbook* [18]. For rating, we considered a study of weak or moderate quality on the EPHPP to have high risk of bias.

## Results

### Study selection

**Fig. 1:**
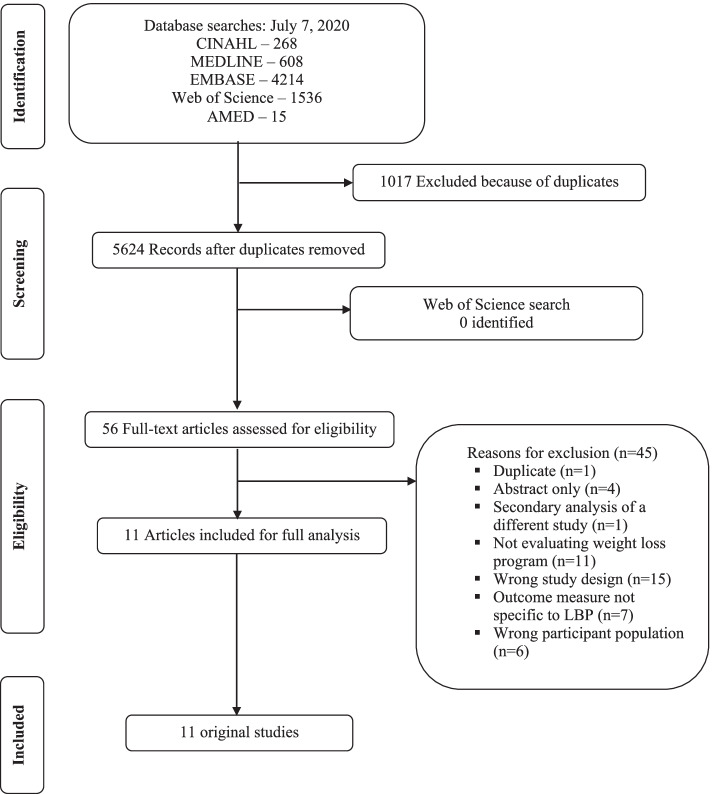
Flow diagram of study selection

The initial electronic database search resulted in a total of 5624 articles after removing duplicates. Following the removal of duplicates and screening of titles and abstracts, 56 full text articles were assessed. Of these articles, 11 fulfilled the inclusion criteria and were included in this review [21–31]. Through additional manual searches of reference lists, hand searches and Web of Science searches, we did not identify any additional eligible studies. Figure 1 shows the flowchart of the inclusion process of this review. A list of all excluded full text studies with reasons for exclusion can be found in Appendix 2. A few potentially eligible studies were excluded because LBP was not an inclusion criterion and it was unclear whether all patients included had LBP at baseline, even when back pain was a primary outcome.

### Study characteristics

All studies included in this systematic review investigated the impact of a weight loss intervention on individuals suffering from LBP. However, most studies did not specify the type of LBP included, with some authors mentioning back pain without further consideration of the specific diagnosis. There was one RCT [30], two NRSIs [28, 29], and eight single-arm studies [21–27, 31]. The outcomes evaluated in these studies were LBP (e.g., Numeric Pain Rating Scale, the presence or absence of pain), disability (e.g., Oswestry Low Back Disability Index) and quality of life (e.g., 36-Item Short Form Health Survey).

### Participants

Across all studies there were 689 participants included, 381 in the nonsurgical intervention studies [27, 28, 30] and 308 in the bariatric surgery studies [21–26, 29, 31]. Study sample sizes ranged from 18 to 175. The mean age ranged from 33 to 57 years old. At baseline, the participants’ average weight ranged from 80.2 kg to 144.5 kg and their baseline BMI ranged from 24.8 kg/m^2^ to 54.2 kg/m^2^. Detailed information on the characteristics of the participants is provided in Table 1.

**Table 1:** Study characteristics

Study Characteristics				
**Study**	**Study Design**	**Study description**	**Inclusion/Exclusion Criteria**	**Participant Information**
Bhandari et al. 2019 [21]	Single-arm	Examination of the effect of weight loss after bariatric surgery on patients with impaired functional ambulatory abilities (bedridden, wheelchair-bound, or walker-dependent)	Inclusion criteria: not reported. Exclusion criteria: patients who had certain gastric lesions, neoplastic findings, family history of gastric cancer, mental health disorders, significant medical co-morbidities precluding sedation, or coagulopathies	34 participants with severe back pain (out of 45 total study participants) were enrolled in this study with 100% follow-up at 1 year. Mean age was 54.7 ± 8.5 yrs. Mean BMI at baseline was 54.2 ± 8.6 kg/m^2^. 27 were walker-dependent, 14 were wheelchair-bound, and 4 were bedridden
Hooper et al. 2007 [22]	Single-arm	Examination of the point prevalence of painful MSK conditions (including LBP) in obese subjects before and after weight loss following bariatric surgery	Inclusion criteria: at least 35y, willing to sign informed consent and able to complete the questionnaires independently Exclusion criteria: subjects who withdrew consent. They were not required to have any MSK conditions to participate	18 participants (1 male) with LBP at baseline (out of 48 total study participants) were enrolled in this study. Mean age was 44 ± 9 yrs. Mean BMI at baseline was 51 ± 8 kg/m^2^
Khoueir et al. 2009 [23]	Single-arm	Assessment of clinically reported changes in chronic axial low back pain symptoms after weight reduction from bariatric surgery morbidly obese subjects	Inclusion criteria: at least 18y with a BMI > 40 kg/m^2^. Patients with a BMI between 35 and 39.9 kg/m2 were also included if they were 50% to 100% more than their ideal weight. They also had to report a two-year history of chronic mechanical low back pain with or without radiculopathy that causes significant disability. Exclusion criteria: not reported	58 consecutive patients were enrolled. Only 38 (30 women) completed both preoperative and postoperative (12 months) questionnaires. All patients had at least a two-year history of chronic mechanical LBP. Mean age was 48.46 ± 10.1 yrs. Mean weight and BMI at baseline were 144.52 ± 41.21 kg and 52.25 ± 12.61 kg/m^2^
Lidar et al. 2012 [24]	Single-arm	Documentation of the effect of significant weight reduction through bariatric surgery in morbidly obese adults on axial back pain, radicular leg pain and quality of life	Not reported	30 morbidly obese patients (15 women) completed the study. Only 25 participated in follow-up at 1 yr. Preoperatively, 26 patients had axial back pain, 16 had radicular leg pain, 15 had both and 4 patients had no axial or radicular pain. Mean age was 49 ± 10.4 yrs. Mean weight and BMI at baseline were 119.6 ± 20.7 kg and 42.8 ± 4.8 kg/m^2^
McGoey et al 1990 [25]	Single-arm	Examination of incidence of chronic pain (including back pain) in an obese population undergoing vertical banded gastroplasty	Not reported	65 participants had, on most days of the month, LBP (62 mechanical, 3 sciatica), which was severe enough to interfere with their activities of daily living (out of 105 total study participants). Mean age was 33.4 years (range 18–58 years). Mean weight at baseline was 125 kg (45 kg overweight)
Melissas et al. 2003 [31]	Single-arm	Assessment of LBP symptoms of morbidly obese candidates before and after vertical banded gastroplasty	Not reported	29 patients experienced LBP symptoms preoperatively (out of 50 total study participants). Mean age was 37.5 ± 10.2 yrs. Mean weight and BMI at baseline were 131.9 ± 25.88 kg 48.03 ± 8.94 kg/m^2^
Melissas et al 2005 [26]	Single-arm	Quantification of the disability caused by LBP in morbidly obese patients and examination of the exact degree of improvement resulting from weight loss following bariatric surgery	Not reported	29 patients (23 female) were enrolled with 100% follow-up at 24 months. All 29 patients had LBP at baseline. Mean age was 37.4 ± 11.2 yrs. Mean weight and BMI at baseline were 132.5 ± 27 kg and 47.2 ± 8.8 kg/m^2^
Roffey et al. 2011 [27]	Single-arm	Assessment of the efficacy of a pilot nonsurgical weight loss program at reducing the severity of LBP in obese adults	Inclusion criteria: referral to a medically supervised nonsurgical weight loss program by primary care physicians; BMI > 30 kg/m^2^; self-reported LBP of any duration and ability to read and write in English Exclusion criteria: inability to participate in the 12-month study period and obesity attributed to a primary endocrine disorder	46 patients (37 female) were enrolled in the study. 40 participants were assessed at week 14 and 34 were assessed at week 53. All participants reported to have experienced LBP at baseline. Mean age was 50.1 ± 12.9 yrs. Mean weight and BMI at baseline were 123.0 ± 25.2 kg and 44.7 ± 7.6) kg/m^2^
Silisteanu et al. 2015 [28]	NRSI	Studying the predictive role of body weight in the emergence and management of CLBP. The control and the experimental group both followed analgesic drug management, physiotherapy, and massage therapy, while the treatment group also underwent a nutritional counselling program	Inclusion criteria: age 18–65 years, LBP with or without radiculopathy disk etiology, and those who completed the evaluation questionnaire and consented to the studies Exclusion criteria: age < 18 years and > 65 years, lumbar pain of another etiology: traumatic, tumor, muscularligamentous, tuberculosis, mental illness, did not complete the evaluation questionnaires and did not sign consent agreement	175 patients (86 women) were enrolled in the study. All patients were diagnosed with CLBP. Baseline BMI was between 24.8 ± 4.8 and 31.1 ± 6.8 Sample size at follow-up, age, and weight at baseline were not reported
Vincent et al. 2012 [29]	NRSI	Examination of whether morbidly obese participants who undergo bariatric surgery demonstrate improvements in joint pain (including back pain) and quality of life compared to nonsurgical counterparts	Not reported	25 participants (21 women) in the bariatric surgery group. Mean LBP score at baseline was 5.2 on the NPRS. Mean age was 41 ± 11 yrs. Mean weight and BMI at baseline were 125 ± 21 kg and 47 ± 7 kg/m^2^ 20 participants (17 women) in the nonsurgical control group. Mean age was 50 ± 7 yrs. Unknown mean LBP score at baseline. Mean weight and BMI at baseline were 115 ± 22 kg and 42 ± 6 kg/m^2^
Williams et al. 2018 [30]	RCT	Assessment of the effectiveness of a 6-month healthy lifestyle intervention on pain in CLBP patients who were overweight or obese. Participants on the waitlist to see an orthopedic surgeon were randomized to receive a healthy lifestyle intervention or waitlist. Intervention included a telephone-based coaching and a telephone advice and clinical consultation	Inclusion criteria: primary complaint of chronic LBP, pain > 3 on a 10 scale or with moderate interference with daily activities, 18y or older, BMI ≥ 27 and < 40 kg/m^2.^ Exclusion criteria: known or suspected serious pathology as the cause of back pain as advised by their general practitioner; previous obesity surgery; currently participating in any prescribed, medically supervised or commercial weight loss program; back surgery in the past 6 months or booked for surgery in the next 6 months; unable to comply with the study protocol that required adaption of meals or exercise due to nonindependent living arrangements; any medical or physical impairment precluding safe participation in exercise, such as uncontrolled hypertension; and unable to speak and read English sufficiently to complete the study procedures	160 participants (95 female) were included (80 intervention, 80 waitlist). One participant was excluded from the intervention group after randomization Intervention group: Mean pain intensity (NPRS) at baseline was 6.7 ± 1.7. Mean age was 56.0 ± 13.3. Mean self-reported weight at baseline was 91.9 ± 16.5 kg. Mean subjective BMI at baseline was 32.4 ± 3.5 Control group: Mean pain intensity (NPRS) at baseline was 6.8 ± 1.6. Mean age was 57.4 ± 13.6. Mean self-reported weight at baseline was 90.8 ± 14.6 kg. Mean subjective BMI at baseline was 32.1 ± 3.6

*LBP* low back pain, *CLBP* chronic low back pain, *BMI* body mass index, *MSK* musculoskeletal, *NPRS* numeric pain rating scale

### Interventions

The one RCT by Williams et al. (2018) evaluated a healthy lifestyle intervention (consisting of telephone-based advice, clinical consultation, and healthy lifestyle coaching) compared to waitlist [30]. Eight of the 11 studies investigated the effect of bariatric weight loss surgery on back pain in individuals with LBP [21–26, 29, 31] (7 single-arm studies, 1 NRSI). One study investigated nutritional and behavioral modification in combination with analgesic drug treatment, physiotherapy, and massage compared to only analgesic drug treatment, physiotherapy, and massage (NRSI) [28]. The last study investigated a multidisciplinary nonsurgical weight loss program (single-arm study) [27].

### Risk of Bias Assessment

The EPHPP Quality Assessment Tool was used to assess the risk of bias in all studies [19]. Ten out of 11 studies received an EPHPP global rating of weak and the only one RCT received a global rating of moderate. Table 2 shows the EPHPP grading process, with primary weaknesses being selection bias, uncontrolled confounders, and lack of blinding.

**Table 2:** Critical appraisal of included studies using the effective public health practice project (EPHPP) quality assessment tool for quantitative studies

**Study**							
	**Selection bias**	**Study design**	**Confounders**	**Blinding**	**Data collection**	**Attrition**	**Global Rating**
Bhandari et al. 2019 [21]	weak	moderate	weak	weak	strong	strong	weak
Hooper et al. 2007 [22]	weak	moderate	weak	weak	strong	strong	weak
Khoueir et al. 2009 [23]	weak	moderate	moderate	weak	strong	moderate	weak
Lidar et al. 2012 [24]	weak	moderate	weak	weak	strong	strong	weak
McGoey et al. 1990 [25]	weak	moderate	weak	weak	weak	moderate	weak
Melissas et al. 2003 [31]	weak	moderate	weak	weak	weak	moderate	weak
Melissas et al. 2005 [26]	weak	moderate	weak	weak	strong	moderate	weak
Roffey et al. 2011 [27]	weak	moderate	weak	weak	strong	weak	weak
Silisteanu et al. 2015 [28]	weak	moderate	weak	weak	strong	weak	weak
Vincent et al. 2012 [29]	weak	moderate	moderate	weak	strong	weak	weak
Williams et al. 2018 [30]	weak	strong	moderate	moderate	strong	moderate	moderate

### Effect of nonsurgical weight loss interventions

#### Randomized controlled trial

Williams et al. randomly assigned 160 participants to a telephone advice session consultation with a 6-month telephone-based healthy lifestyle coaching service versus waitlist and followed them for 26 weeks [30]. The results of the study show that the healthy lifestyle intervention did not improve back pain intensity (MD = 0.3, 95% CI -0.4 to 1.0), decrease disability (MD = -0.1, 95% CI -1.7 to 1.5) or improve quality of life (Physical function: MD = -0.6, 95% CI -3.5 to 2.4; Mental function: MD = -1.7, 95% CI -5.4 to 2.0) for patients with LBP who were overweight/obese. Therefore, there is low-quality evidence given there is one moderate quality study (GRADE reduced due to risk of bias) that a lifestyle intervention is no better than waitlist at improving pain, disability, and quality of life in patients with LBP. See Table 3 for detailed results.

**Table 3:** Pain, disability, and quality of life outcomes from the RCT (Williams et al. 2008 [30])

Effect on Pain, Disability, or Quality of Life					
	**Type of Intervention**	**Outcome**	**Within Group Difference (MD, 95% CI)**	***P*-Value**	**Between group difference (MD, 95% CI)**
Pain outcomes	Telephone based advice, clinical consultation and healthy lifestyle coaching	NPRS (baseline to 2, 6, 10, 14, 18, 22 and 26 weeks)	Week 2: 0.0 (-0.6 to 0.6) Week 6: -0.1 (-0.8 to 0.5) Week 10: 0.6 (0.0 to 1.3) Week 14: 0.4 (-0.2 to 1.1) Week 18: 0.8 (0.2 to 1.5) Week 22: 0.4 (-0.3 to 1.1) Week 26: 0.08 (-0.04 to 0.21)	= 1.00 = 0.72 = 0.05 = 0.20 = 0.01 = 0.24 = 0.36	Week 2: 0.0 (-0.6 to 0.6) Week 6: -0.1(-0.8 to 0.5) Week 10: 0.6 (0.0 to 1.3) Week 14: 0.4 (-0.2 to 1.1) Week 18: 0.8 (0.2 to 1.5) Week 22: 0.4 (-0.3 to 1.1) Week 26: 0.3 (-0.4 to 1.0) Week 2 P = 1.00 Week 6 P = 0.72 Week 10 P = 0.05 Week 14 P = 0.20 Week 18 P = 0.01 Week 22 P = 0.24 Week 26 P = 0.36
	Control	NPRS (baseline to 2, 6, 10, 14, 18, 22 and 26 weeks)	Week 2: -0.4 (-0.9 to 0.1) Week 6: -0.6 (-1.2 to 0) Week 10: -0.4 (-1 to 0.2) Week 14: 0.0 (-0.5 to 0.5) Week 18: -0.3 (-0.8 to 0.2) Week 22: -0.6 (-1.2 to 0) Week 26: -0.5 (-1.1 to 0.1)	Not reported	
Disability outcomes	Telephone based advice, clinical consultation and healthy lifestyle coaching	RMDQ (baseline to 6 and 26 weeks)	Week 6: 0.8 (-0.6 to 2.2) Week 26: -0.1 (-1.7 to 1.5)	< 0.05	Week 6: 0.8 (-0.6 to 2.2) P value not reported
	Control	RMDQ (baseline to 6 and 26 weeks)	Week 6: 0.0 (-1.6 to 1.6) Week 26: -1.1 (-3 to 0.8)	Not reported	Week 26: -0.1 (-1.7 to 1.5) P value not reported
Quality of life outcomes	Telephone based advice, clinical consultation and healthy lifestyle coaching	SF12.v2 Physical function (Baseline to 6 and 26 weeks)* SF12.v2 Mental function (baseline to 6 and 26 weeks)*	Week 6: 0.5 (-2.6 to 3.6) Week 26: 0.8 (-2.9 to 4.5) Week 6: -0.1 (-4.4 to 4.2) Week 26: -0.2 (-5.4 to 5)	Not reported	SF12.v2 Physical function: Week 6 = -0.3 (-3.0 to 2.4) Week 26 = -0.6 (-3.5 to 2.4)
	Control	SF12.v2 Physical function (Baseline to 6 and 26 weeks) SF12.v2 Mental function (baseline to 6 and 26 weeks)	Week 6: 1.1 (-2.2 to 4.4) Week 26: 1.3 (-2 to 4.6) Week 6: -1.1 (-5.2 to 3) Week 26: -1.8 (-6.3 to 2.7)	Not reported	SF12.v2 Mental function: Week 6 = -0.9 (-4.3 to 2.4) Week 26 = -1.7 (-5.4 to 2.0)

*NPRS* numeric pain rating scale, *MD* mean difference, *CI* confidence interval, *RMDQ* roland morris disability questionnaire, *MD* mean difference, *CI *confidence interval, *SF12.v2* short form health survey version 2, *MD* mean difference, *CI* confidence interval. *Sample size not reported

#### Non-randomized study of intervention

Silisteanu et al. conducted a NRSI that allocated 175 patients diagnosed with chronic LBP to the control (analgesic drug treatment, physiotherapy, and massage) and treatment groups (same as control plus nutritional counselling and physical activity) [28]. The study demonstrated that when nutritional counselling was applied, the VAS pain index (*p* < 0.01) and QOLS (*p* < 0.05) (except for men in urban areas) were further improved in the treatment group compared to the control group. Mean differences and confidence intervals could not be calculated as sample size was not available for each group. Although the authors state that correlation analysis demonstrated strong associations between BMI, VAS and QOLS following nutritional intervention, they did not provide results to allow a better interpretation of the findings. See Appendix 3 for weight loss results. Therefore, there is very low-quality evidence from one weak quality study that a nutritional and behavioural modification treatment program may be superior to controls at improving pain and quality of life in obese patients with LBP. See Tables 4 and 5 for detailed results.

**Table 4:** Pain outcomes from non-RCT studies

Effect on Pain				
**Study**	**Type of Intervention**	**Outcome**	**Within group difference (MD, 95% CI)**	***P*-Value**
Bhandari et al. 2019 [21]	Bariatric surgery	NPRS (baseline to 1 year)	MD = -5.0, 95% CI -5.7 to -4.3	< 0.001
Hooper et al. 2007 [22]	Gastric bypass surgery	Frequency of L-spine symptoms before surgery Frequency of L-spine symptoms after surgery (6–12 months)	*n* = 18 (38%) *n* = 3 (6.25%) OR = 0.11, 95% CI 0.03 to 0.41	Pre vs post < 0.001
Khoueir et al. 2009 [23]	Bariatric surgery	VAS (baseline to 12 months)	MD = -2.3, 95% CI -3.8 to -0.8	< 0.006
Lidar et al. 2012 [24]	Bariatric surgery	VAS axial back pain (baseline to 12 months) VAS leg pain (baseline to 12 months)	MD = -4.4, 95% CI -5.9 to -2.5 MD = -3.0, 95% CI -4.5 to -1.5	< 0.001 < 0.001
McGoey et al. 1990 [25]	Bariatric surgery	Standardized pain questionnaire (baseline to 22.5 months)	Reduction in participants experiencing pain from 62% preoperatively to 11% postoperatively (*n *= 104) OR = 0.07, 95% CI 0.04 to 0.15	
Melissas et al. 2003 [31]	Vertical banded gastroplasty	Complete resolution of LBP (2 years post-op) Significant improvement in LBP (2 years post-op)	19/29 pts who suffered from LBP preoperatively 10/29 pts who suffered from LBP preoperatively	Not reported
Melissas et al. 2005 [26]	Vertical banded gastroplasty	VAS – pain immediately (baseline to 24 months) VAS – pain at its worst pattern (baseline to 24 months) VAS—at its best pattern (baseline to 24 months)	MD = -1.3, 95% CI -2 to -0.6 MD = -3.4, 95% CI -4.4 to -2.4 MD = -0.7, 95% CI -1.1 to -0.3	< 0.001 < 0.001 = 0.006
Roffey et al. 2011 [27]	Nonsurgical weight loss program	NPRS (baseline to 14 weeks)	MD = -1.6, 95% CI -2.6 to -0.6	= 0.001
		NPRS (baseline to 53 weeks)	MD = -0.7, 95% CI -1.8 to 0.4	= 0.07
Silisteanu et al 2015 [28]	Nutritional and behavioral modification	VAS (beginning and end of each of the 3 rehabilitation programs)	Men URBAN: Initial: 6.0 ± 1.4 | Final: 3.0 ± 0.7 Men RURAL: Initial: 7.0 ± 1.1 | Final: 3.5 ± 0.8 Women URBAN: Initial: 7.0 ± 1.3 | Final: 4.0 ± 0.9 Women RURAL: Initial: 7.0 ± 1.2 | Final: 4.0 ± 0.8	Btw pre-treatment and post-treatment *P* < 0.01
	Control	VAS (beginning and end of each of the 3 rehabilitation programs)	Men URBAN: Initial: 7.0 ± 1.5 | Final: 5.0 ± 1.5 Men RURAL: Initial: 6.0 ± 1.5 | Final: 4.0 ± 1.5 Women URBAN: Initial: 7.0 ± 1.4 | Final: 5.0 ± 1.4 Women RURAL: Initial: 6.0 ± 1.4 | Final: 4.0 ± 1.5	Btw pre-treatment and post-treatment *P* < 0.01
Vincent et al. 2012 [29]	Bariatric surgery	NPRS (baseline to 3 months)	No moderate to severe LBP at baseline = 25.0% No moderate to severe LBP at 3 months = 61.1% OR = 4.75, 95% CI 1.41 to 16.05	N/A
	Nonsurgical control	NPRS (baseline to 3 months)	No values reported. “The control group did not demonstrate any significant changes in joint pain”	N/A

*NPRS* numeric pain rating scale, *OR* odds ratio, *VAS* visual analogue scale, *MD* mean difference, *CI* confidence interval

**Table 5:** Quality of life outcomes from non-RCT studies

Effect on Quality of Life							
**Study**	**Type of Intervention**		**Outcome**		**Within group difference (MD, 95% CI)**		***P*-Value**
Khoueir et al. 2009 [23]	Bariatric surgery		SF-36 Physical function (baseline to 12 months) SF-36 Mental health (baseline to 12 months)		MD = 25.7, 95% CI 15.1 to 36.4 MD = 3.4, 95% CI -1 to 7.8		< 0.0001 = 0.03
Lidar et al. 2012 [24]	Bariatric surgery		SF-36 Physical function (baseline to 12 months) SF-36 Mental function (baseline to 12 months) MA (baseline to 12 months)		No significant changes were noted when comparing pre-operative with post-operative data for both the mental and physical components MD = 2.0, 95% CI 1.3 to 2.7		= 0.097 = 0.104 P < 0.001
Silisteanu et al. 2015 [28]	Nutritional and behavioral modification		QOLS (beginning and end of each of the 3 rehabilitation programs)		Men URBAN: Initial: 0.6 ± 0.09 | Final: 0.8 ± 0.06 Men RURAL: Initial: 0.6 ± 0.1 | Final: 0.7 ± 0.07 Women URBAN: Initial: 0.6 ± 0.06 | Final: 0.8 ± 0.06 Women RURAL: Initial: 0.6 ± 0.09 | Final: 0.8 ± 0.06		Sig. of diff. btwn pre- and post-treatment P < 0.05 except for men in the urban area
	Control		QOLS (beginning and end of each of the 3 rehabilitation programs)		Men URBAN: Initial: 0.5 ± 0.08 | Final: 0.5 ± 0.09 Men RURAL: Initial: 0.5 ± 0.08 | Final: 0.7 ± 0.09 Women URBAN: Initial: 0.5 ± 0.07 | Final: 0.7 ± 0.09 Women RURAL: Initial: 0.5 ± 0.08 | Final: 0.6 ± 0.09		Sig. of diff. btwn pre- and post-treatment P < 0.05 except for men in the urban area
Vincent et al. 2012 [29]	Bariatric surgery		SF-36 Physical function* (baseline to 3 months) SF-36 Mental function (baseline to 3 months)		Physical component of SF-36 Baseline: 32.8 ± 10.1 Month 3: 44.6 ± 10.6 Mental component of SF-36 Baseline: 44.4 ± 10.5 Month 3: 50.0 ± 10.7		Not reported
	Nonsurgical control		SF-36 Physical function* (baseline to 3 months) SF-36 Mental function (baseline to 3 months)		Physical component of SF-36 Baseline: 26.7 ± 5.4 Month 3: 26.7 ± 5.2 Mental component of SF-36 Baseline: 48.6 ± 11.3 Month 3: 48.4 ± 11.3		Not reported
		Between group difference (bariatric surgery vs nonsurgical control)				Between group SF-36 Physical function MD = 17.9, 95% CI 12.7 to 23.1 Between group SF-36 Mental function MD = 1.6, 95% CI -5.0 to 8.2	

*SF-36* 36-item short form health survey, *MA* mMoorehead-ardelt, *QOLS* quality of life scale, *MD* mean difference, *CI* confidence interval. * Calculated based on baseline sample size, as no sample size was reported for follow-up and assuming no drop-out

### Single-arm studies

Roffey et al. conducted a pilot study evaluating a 52-week multidisciplinary weight loss program in 46 obese adults [27]. At week 14, of the 98% of participants that lost more than 5% of their body weight, 50% of participants reported clinically significant improvements in back pain (Minimal Clinically Important Difference (MCID) NPRS = 2/10) (MD = -1.6, 95% CI -2.6 to -0.6) and 73% of participants reported clinically significant improvements in disability (MCID ODI = 10/50) (MD = -8.4, 95% CI -16.2 to -0.06). The results of this study also demonstrated that participants who continued to lose weight beyond 14 weeks and had achieved a greater percentage reduction in BMI after one year, had a positive correlation with improvement in LBP and ODI scores at one year. Thus, given that there is one weak quality study, there is very low-quality evidence that back pain and disability can be reduced after a multidisciplinary weight loss program. See Tables 4 and 6 for detailed results.

**Table 6:** Disability outcomes from non-RCT studies

Effect on Disability				
**Study**	**Type of Intervention**	**Outcome**	**Within group difference (MD, 95% CI)**	***P*-Value**
Khoueir et al. 2009 [23]	Bariatric surgery	ODI (baseline to 12 months)	MD = -6.3, 95% CI -14.2 to 1.6	= 0.05
Melissas et al. 2005 [26]	Vertical banded gastroplasty	RMDQ (baseline to 24 months) ODI (baseline to 24 months) Waddell Disability Index (baseline to 24 months)	MD = -6.0, 95% CI -8 to -4 MD = -15.6, 95% CI -21.9 to -9.3 MD = -2.3, 95% CI -2.8 to -1.7	< 0.001 < 0.001 < 0.001
Roffey et al. 2011 [27]	Nonsurgical weight loss program	ODI (baseline to14 weeks)	MD = -8.4, 95%CI -16.2 to—0.06	= 0.0005
		ODI (baseline to 52 weeks)	MD = -4.8, 95%CI -13.7 to—0.06	= 0.0009

*ODI* oswestry low back disability index, *RMDQ* roland morris disability questionnaire, *MD* mean difference, *CI* confidence interval

### Effect of surgical weight loss interventions

#### Non-randomized study of intervention

Vincent et al. compared a bariatric surgery group to nonsurgical counterparts and identified that at 3 months follow up, 61.1% (*n* = 25) of participants in the bariatric group had no moderate to severe LBP compared to 25% at baseline (OR = 4.8, 95% CI 1.4 to 16.1), while the control group did not demonstrate any significant changes from baseline [29]. This study also demonstrated between group differences in the SF-36 physical component score of MD = 17.9, 95% CI 12.7 to 23.1 but no difference between groups for the mental component score of MD = 1.6, 95% CI -5.0 to 8.2. Therefore, there is very low-quality evidence from a single weak quality study that bariatric surgery compared to no surgery may improve pain and quality of life (physical component only) at 3 months follow up. See Tables 4 and 5 for detailed results.

### Single-arm studies

Seven single-arm studies of weak quality assessed the effectiveness of bariatric surgery on LBP, disability, and quality of life at a median of 12 months after surgery (range 12 – 24 months) [21–26, 31]. Six studies evaluated back pain reduction (Numeric pain rating scale (NPRS) and Visual analogue scale (VAS)) at long-term (12 to 24 months) with changes from baseline ranging from -5.0 to -0.7 (on a scale from 0 to 10). Bhandari et al. reported a moderate association between BMI change and NPRS of back pain 1 year post operation (*r* = 0.40; *P* = 0.002), while Lidar et al. reported no significant correlation between decreased BMI and improvement in back pain (*r* = 0.231; *P* = 0.218). See Appendix 3 for weight loss results. Melissas et al. (2003) reported that at 24 months, 66% (*n* = 19/29) of patients had complete resolution of LBP and 34% (*n* = 10/29) of patients reported improvement in LBP symptoms [31]. McGoey et al. demonstrated a 51% (*n* = 53/104) reduction in the number of participants experiencing LBP at 22.5 months follow-up [25]. The authors state that back pain relief was not statistically different between patients who lost a low to moderate amount of weight (< 27 kg) compared with those who lost a large amount of weight (> 45 kg). However, they did not provide results to allow a better interpretation of the findings. These results from single-arm studies of weak quality, demonstrate very low-quality evidence that bariatric surgery may lead to a reduction in LBP in the long-term.

There were two single-arm studies that demonstrated statistically significant effects of weight loss surgery on disability (ODI) at long-term (12 to 24 months) [23, 26] with change from baseline ranging from -15.6 to -6.3 (on a scale from 0 to 100). These studies demonstrate very low-quality evidence from single-arm studies of weak quality that bariatric surgery may improve disability at long-term.

Lastly, there was one single-arm study that reported a statistically significant improvement and possible clinically significant change in quality of life (SF-36 Physical Function) at long-term (12 months) (MD = 25.7, 95% CI 15.1 to 36.4) [23]. However, another study [24] reported no significant changes in both the mental and physical components of SF-36 at 12 months. Therefore, there is conflicting evidence from two weak quality studies on the effect of bariatric surgery on quality of life at long-term. See Tables 4, 5 and 6 for detailed results.

### Overall quality of evidence rating

Due to the fact that all papers, except one, were non-RCTs and had high risk of bias and small sample sizes, the overall quality of the body of evidence for all intervention types and outcomes are rated as very low-quality, as described above.

## Discussion

This systematic review included 11 studies evaluating weight loss programs to improve self-reported outcomes of LBP. Most of the studies included were single-arm studies of surgical interventions on obese participants. Of the included studies, there was one moderate quality RCT, two weak quality NRSIs and eight weak quality single-arm studies. There was low-quality evidence that a lifestyle intervention is not better than wait list in improving back pain, disability, and quality of life in patients with LBP. There was very low-quality evidence that nutritional and behavioural modification may be superior to controls at improving back pain and quality of life in obese participants with LBP. Further, there was very low-quality evidence that bariatric surgery may improve back pain and disability in obese participants. Our study demonstrates a lack of high-quality studies in the literature investigating the effectiveness of weight loss programs for LBP.

A multitude of weight loss interventions are available and the most appropriate conservative method for patients with back pain or chronic back pain is yet to be established. Importantly, compliance with weight loss interventions is usually poor and even when an individual losses weight with a program, maintenance is usually a problem [32]. In fact, one study noted that the positive effects of weight loss were reversed at follow-up likely due to a subsequent weight gain of participants [27]. Thus, weight loss interventions may be susceptible to adherence issues and outcomes may be dependent on the maintenance of weight loss. Future studies evaluating the amount of weight loss necessary to observe improvements in back pain and disability and mediators of change are needed. Additionally, future studies should also evaluate the effect of amount of weight loss on other types of joint pain, such as knee osteoarthritis, to determine how weight loss can affect an individual in various aspects.

Potential clinically significant effects of weight loss surgery on LBP were found across eight studies of weak quality [21–27, 31]. McGoey found that back pain relief was not superior in patients who lost a large amount of weight (> 45 kg) compared to those who lost a low to moderate amount of weight (< 27 kg) [25]. Thus, patients may even benefit with clinically significant improvements in pain from a modest reduction in weight loss [33]. However, more evidence is needed to evaluate the relationship between weight loss and symptom reduction as this relationship may not be linear with the possibilities of plateauing. Furthermore, these results suggest that other indirect benefits of weight loss could be the primary mediator in the reduction of LBP such as increased mobility, increased physical activity level and changes in psychosocial factors such as self-esteem and self-efficacy. In fact, many of the included full text studies were secondary analysis of weight loss interventions, for which reduction of LBP was not the primary objective. Conversely, weight loss can also be a consequence of LBP treatments such as physical activity and medications [34, 35]. More studies with a primary focus on weight loss interventions for the management of LBP are needed. Studies should focus on the effectiveness of the intervention but also on the amount of weight lost required for symptom improvement, subgroups of BMI that require a weight loss intervention as well as long term adherence to these programs.

There is a lack of literature examining the effects of weight loss programs on LBP despite the large number of studies identifying obesity or high BMI as important risk factors for the development of LBP. This review shows that studies investigating the effect of a weight loss intervention on LBP have been of extremely weak methodological quality with high risk of bias. Although the results of the studies included in this review are promising, no definitive conclusions can be drawn at this time due to the paucity of high-quality RCTs.

Some limitations of this review include the low-quality of studies evaluating within group effects, inconsistent use of outcome measures, and the differences in implementation of the interventions. Most of the included studies are single-arm studies with poor quality of reporting—particularly the inclusion criteria, patient characteristics, and results. Additionally, the inclusion of multiple types of interventions in this review make it difficult to form any solid conclusions and do not allow for pooling of data. However, we presented the results separately by intervention (i.e., bariatric surgery vs nonsurgical weight loss interventions). Lastly, the exclusion of grey literature is a limitation of our review and a deviation from the Cochrane Handbook.

The results of this review highlight the scarcity of research examining the effect of weight loss on LBP. It demonstrates that although associations have been drawn between weight and LBP, there is only very low-quality evidence that some weight loss interventions lead to improvements in LBP and disability. Further, there is evidence to suggest that adherence to these interventions is problematic, particularly in the long term. Future research should focus on conducting higher quality trials that evaluate the effect of weight loss on improving outcomes such as LBP, disability, and quality of life in individuals with LBP with considerations on mediators and moderators of outcomes.

## Supplementary Information

**Additional file 1. **

## Data Availability

All data generated or analysed during this study are included in this published article and its supplementary information files.

## Abbreviations

BMI: Body mass index
EPHPP: Effective Public Health Practice Project
GRADE: Grading of Recommendations Assessment, Development and Evaluation
LBP: Low back pain
MD: Mean difference
NPRS: Numeric pain rating scale
NRSI: Non-randomized studies of intervention
RCT: Randomized controlled trials
VAS: Visual analogue scale
