# Supplementary material for: The effectiveness of weight loss programs for low back pain: a systematic review
Source: BMC Musculoskelet Disord. 2022 May 23;23:488. doi: 10.1186/s12891-022-05391-w (PMC9125929; doi:10.1186/s12891-022-05391-w)
Supplement: Supplementary file 1 — Additional file 1. [file 12891_2022_5391_MOESM1_ESM.docx]

**Appendix 1 - Search Strategy**

Across all databases, no specific limits were placed (i.e., language, type of study, etc.).

**CINAHL (1937 to June 2020)**

S1 (MH “low back pain”)

S2 (MH “spinal stenosis”)

S3 (MH “Back Pain+”)

S4 (MH “thoracic vertebrae”)

S5 (MH “radiculopathy”)

S6 (MH “Spondylolisthesis”)

S7 (MH “Spondylolysis+”)

S8 (MH “Spondylosis+”)

S9 (MH “Sciatica”)

S10 (MH “coccydynia”)

S11 (MH “intervertebral disc displacement”)

S12 (MH “Polyradiculopathy+”)

S13 (MH “Spinal nerve roots+”)

S14 “back ache”

S15 “backache”

S16 “back pain”

S17 “lumbago”

S18 “sciatic*”

S19 “ischias”

S20 “ischialgia”

S21 “lumboischialgia”

S22 “radiculalgia”

S23 “radiculopathy*”

S24 “radiculi*”

S25 “low back pain”

S26 “dorsalgia”

S27 “lumbar pain”

S28 “lumbar stenosis”

S29 “disc herniation”

S30 “spin* stenos*”

S31 “spondylolysis”

S32 “spondylosis”

S33 “lumbar radiculopathy”

S34 “Spinal nerve root”

S35 Or/S1-S34

S36 (MH "Weight Loss+")

S37 (MH “Weight Reduction Programs”)

S38 “Fat loss”

S39 “body fat reduction”

S40 “body mass reduction”

S41 “body mass index reduction”

S42 “weight loss”

S43 “weight reduc*”

S44 OR/S36-S43

S45 S35 AND S44

S46 (MH “Human”)

S47 (MH “Animals”)

S48 S47 NOT S46

S49 S45 not S48

**Results: 264**

**MEDLINE (1946 to June 2020)**

1. low back pain/
2. sciatica/
3. intervertebral disc displacement/
4. exp spinal nerve roots/
5. spinal stenosis/
6. radiculopathy/
7. exp spondylitis/
8. exp spondylosis/
9. polyradiculopathy/
10. low back pain.mp.
11. back ache.mp.
12. backache.mp.
13. back pain.mp.
14. dorsalgia.mp.
15. lumbago.mp.
16. lumbar pain.mp.
17. lumbar stenosis.mp.
18. disc herniation.mp.
19. sciatic*.mp.
20. ischias.mp.
21. ischialgia.mp.
22. lumboischialgia.mp.
23. radiculalgia.mp.
24. spin* stenos*.mp.
25. coccydynia.mp.
26. spondylolysis.mp.
27. spondylosis.mp.
28. radiculitis.mp.
29. lumbar radiculopathy.mp.
30. or/ 1-29
31. exp Weight Loss/
32. Weight Reduction Programs/
33. Fat loss.mp.
34. body fat reduction.mp.
35. body mass reduction.mp.
36. body mass index reduction.mp.
37. weight loss.mp.
38. weight reduc*.mp.
39. or/ 29-36
40. 28 and 37
41. Humans/
42. Animals/
43. 40 not 39
44. 38 not 41

**Results: 606**

**EMBASE (1974 to June 2020)**

1. low back pain/
2. intervertebral disk hernia/
3. exp backache/
4. exp vertebral canal stenosis/
5. exp radiculopathy/
6. exp “spinal root”/
7. exp spondylitis/
8. exp spondylosis/
9. polyradiculopathy.mp.
10. low back pain.mp.
11. back ache.mp.
12. backache.mp.
13. back pain.mp.
14. dorsalgia.mp.
15. lumbago.mp.
16. lumbar pain.mp.
17. lumbar stenosis.mp.
18. disc herniation.mp.
19. sciatic*.mp.
20. ischias.mp.
21. ischialgia.mp.
22. lumboischialgia.mp.
23. radiculalgia.mp.
24. radiculopathy*.mp.
25. spin* stenos*.mp.
26. radiculitis*.mp.
27. coccydynia.mp.
28. sciatic*.mp.
29. spondylosis.mp.
30. spondylolysis.mp.
31. lumbar radiculopathy.mp.
32. OR/1-31
33. exp body weight loss/
34. weight loss program/
35. Fat loss.mp.
36. Body fat reduction.mp.
37. Body mass reduction.mp.
38. Body mass index reduction.mp.
39. Weight loss.mp.
40. Weight reduc*.mp.
41. OR/ 33-40
42. 32 AND 41
43. Human/
44. Animal/
45. 44 NOT 43
46. 42 NOT 45

**Results: 4209**

**Web of Science (1976 to June 2020)**

1. ALL=(low back pain)
2. ALL=(Sciatica)
3. ALL=(intervertebral disc displacement)
4. ALL=(spinal nerve roots)
5. ALL=(spinal stenosis)
6. ALL=(radiculopathy)
7. ALL=(spondylitis)
8. ALL=(spondylosis)
9. ALL=(polyradiculopathy)
10. ALL=(back ache)
11. ALL=(backache)
12. ALL=(back pain)
13. ALL=(dorsalgia)
14. ALL=(lumbago)
15. ALL=(lumbar pain)
16. ALL=(lumbar stenosis)
17. ALL=(disc herniation)
18. ALL=(sciatica)
19. ALL=(ischias)
20. ALL=(ischialgia)
21. ALL=(lumboischialgia)
22. ALL=(radiculalgia)
23. ALL=(spinal stenosis)
24. ALL=(coccydynia)
25. ALL=(spondylolysis)
26. ALL=(radiculitis)
27. ALL=(lumbar radiculopathy)
28. Or/#1-#27
29. ALL=(weight loss)
30. ALL=(weight loss program)
31. ALL=(weight reduction program)
32. ALL=(fat loss)
33. ALL=(body fat reduction)
34. ALL=(body mass reduction)
35. ALL=(body mass index reduction)
36. ALL=(weight reduction)
37. Or/#29-#36
38. #27 and #28
39. ALL=(human)
40. ALL=(animals)
41. #40 not #39
42. #38 not #41

**Results: 1534**

**AMED (1985 to June 2020)**

1. Low back pain/
2. Sciatica/
3. Spinal nerve roots/
4. Spinal stenosis/
5. Exp spondylitis/
6. Low back pain.mp.
7. Back ache.mp.
8. Backache.mp.
9. Back pain.mp.
10. Dorsalgia.mp.
11. Lumbago.mp.
12. Lumbar pain.mp.
13. Lumbar stenosis.mp.
14. Disc herniation.mp.
15. Sciatic*.mp.
16. Ischias.mp.
17. Ischialgia.mp.
18. Lumboischialgia.mp.
19. Coccydynia.mp.
20. Spondylolysis.mp.
21. Spondylosis.mp.
22. Radiculitis.mp.
23. Lumbar radiculopathy.mp.
24. Or/ 1-23
25. Exp weight loss/
26. Weight reduction program.mp.
27. Fat loss.mp.
28. Body fat reduction.mp.
29. Body mass reduction.mp.
30. Body mass index reduction.mp.
31. weight loss.mp.
32. Or/ 25-31
33. 24 and 32
34. Humans/
35. Animals/
36. 35 not 34
37. 33 not 36

**Results: 15**

**Appendix 2 - Excluded Trials**

**Appendix 2 Table.** Reason for excluding potentially eligible studies.

| **Study** | **Reason for Exclusion** |
| --- | --- |
| Abu MA, Ghani NAA, Shan LP, Sulaiman AS, Omar MH, Ariffin MHM, et al. Do exercises improve back pain in pregnancy? Horm Mol Biol Clin Investig. 2017;32(3 PG-7):7. | Not evaluating weight loss program |
| Ahroni JH, Montgomery KF, Watkins BM. Laparoscopic adjustable gastric banding: weight loss, co-morbidities, medication usage and quality of life at one year. Obes Surg [Internet]. 2005;15(5 PG-641–7):641–7. | Outcome measure not specific to LBP |
| A.J. W, J. W, K.M. O, L. W, S. Y, Williams C M. A telephone-based lifestyle behavioural intervention for overweight or obese patients with low back pain. Obes Rev [Internet]. 2016;17(SUPPL. 2 PG-156):156. | Abstract only |
| Andersen LN, Juul-Kristensen B, Sorensen TL, Herborg LG, Roessler KK, Sogaard K. Efficacy of Tailored Physical Activity or Chronic Pain Self-Management Programme on return to work for sick-listed citizens: A 3-month randomised controlled trial. Scand J Public Heal. 2015;43(7 PG-694–703):694–703. | Not evaluating weight loss program |
| Arranz LI. Effects of Obesity on Function and Quality of Life in Chronic Pain. Nutritional Modulators of Pain in the Aging Population. London: Academic Press Ltd-Elsevier Science Ltd; 2017. 151–170 p. | Wrong study design |
| Arranz LI, Rafecas M, Alegre C. Effects of Obesity on Function and Quality of  Life in Chronic Pain Conditions. Curr Rheumatol Rep. 2014;16(1 PG-8):8. | Not evaluating weight loss program |
| Baena-Beato PA, Delgado-Fernandez M, Artero EG, Robles-Fuentes A, Gatto-Cardia MC, Arroyo-Morales M. Disability Predictors in Chronic Low Back Pain After Aquatic Exercise. Am J Phys Med Rehabil. 2014;93(7 PG-615–623):615–23. | Not evaluating weight loss program |
| Bloxham SR, Layden J, Jane B, Peers C, Scragg S. The longitudinal effects of a physical activity programme on the physical fitness and disability of back pain patients: Service evaluation. J Back Musculoskelet Rehabil. 2020;33(1 PG-7–13):7–13. | Not evaluating weight loss program |
| Bowerman S, Bellman M, Saltsman P, Garvey D, Pimstone K, Skootsky S, et al. Implementation of a primary care physician network obesity management program. Obes Res. 2001;9(PG-321S-325S):321S-325S. | Outcome measure not specific to LBP |
| C. V, Nascimento L. Core stabilization training as a way to relieve a low back pain and increase the lumbar spine stability in obese patients using vlcd. Obes Facts [Internet]. 2012;5(SUPPL. 1 PG-66):66. | Not evaluating weight loss program |
| Coodley EL, Conston D, Xavier A, Glossbrenner D, Andrews C, King W, et al. PROBLEM – LOW-BACK-PAIN, WEIGHT-LOSS, AND WEAKNESS. Emerg Med. 1986;18(3 PG-85-):85-. | Wrong study design |
| Çakır T, Oruç MT, Aslaner A, Duygun F, Yardımcı EC, Mayir B, et al. The effects of laparoscopic sleeve gastrectomy on head, neck, shoulder, low back and knee pain of female patients. Int J Clin Exp Med. 2015;8(2):2668–73. | Wrong participant population |
| Daentzer D, Hohls T, Noll C. Has overweight any influence on the effectiveness of conservative treatment in patients with low back pain? Eur Spine J. 2015;24(3 PG-467–473):467–73. | Not evaluating weight loss program |
| Dixon JB, Dixon ME, O’Brien PE. Quality of life after lap-band placement: influence of time, weight loss, and comorbidities. Obes Res [Internet]. 2001;9(11 PG-713–21):713–21. | Outcome measure not specific to LBP |
| Endo T, Abe T, Akai K, Kijima T, Takeda M, Yamasaki M, et al. Height loss but not body composition is related to low back pain in community-dwelling elderlies: Shimane CoHRE study. BMC Musculoskelet Disord. 2019;20(PG-7):7. | Wrong study design |
| Evers Larsson U. Influence of weight loss on pain, perceived disability and observed functional limitations in obese women. Int J Obes [Internet]. 2004;28(2 PG-269–277):269–77. | Wrong study design |
| F. D-P, Fouquet B. Obesity, weight reduction and low back pain. Rev du Rhum Monogr [Internet]. 2016;83(1 PG-50–55):50–5. | Wrong study design |
| Gallart-Aragón T, Fernández-Lao C, Galiano-Castillo N, Cantarero-Villanueva I, Lozano-Lozano M, Arroyo-Morales M. Improvements in health-related quality of life and pain: A cohort study in obese patients after laparoscopic sleeve gastrectomy. J Laparoendosc Adv Surg Tech. 2018;28(1):53–7. | Wrong participant population |
| Gandhi SD, Radcliff KE. Obesity in lumbar spine surgery. Curr Orthop Pr. 2016;27(2 PG-135–139):135–9. | Wrong study design |
| G. M, C. D, E. Om, C. B, C. B, K. G, et al. Impact of a multidisciplinary weight management service on musculoskeletal pain in obese individuals. Obes Facts [Internet]. 2017;10(Supplement 1 PG-182):182. | Wrong study design |
| Hooper M M. Tending to the musculoskeletal problems obesity. Cleve Clin J Med [Internet]. 2006;73(9 PG-839–845):839–45. | Wrong study design |
| H.K. V, K.B. D, B. C, K.M. L, Seay A. Gait, musculoskeletal pain, and quality of life can be favorably modified by surgery-induced weight loss. PM R [Internet]. 2011;3(10 SUPPL. 1 PG-S243):S243. | Abstract only |
| Is weight loss an effective treatment for back pain?...second of a two-part article. Back Lett [Internet]. 2006 Feb;21(2):13–22. | Wrong study design |
| Josbeno DA, Jakicic JM, Hergenroeder A, Eid GM. Physical activity and physical function changes in obese individuals after gastric bypass surgery. Surg Obes Relat Dis. 2010;6(4):361–6. | Wrong participant population |
| Kaukua J, Pekkarinen T, Sane T, Mustajoki P. Health-related quality of life in obese outpatients losing weight with very-low-energy diet and behaviour modification: a 2-y follow-up study. Int J Obes. 2003;27(9 PG-1072–1080):1072–80. | Outcome measure not specific to LBP |
| Khoueir P, Black MH, Crookes PF, Kaufman HS, Katkhouda N, Wang MY.  Prospective assessment of axial back pain symptoms before and after bariatric  weight reduction surgery. J Neurosurg. 2008;108(4 PG-871–871):A871–A871. | Duplicate |
| Kim B, Kim S. The influence of weight reduction programs consisting of caloric restriction and/or exercise on the vital age of men with obesity. Arch Budo. 2019;15(PG-139-148):139–48. | Outcome measure not specific to LBP |
| Kotowski SE, Davis KG. Influence of weight loss on musculoskeletal pain: Potential short-term relevance. Work. 2010 ;36(3) :295–304. | Wrong participant population |
| Koulischer S, Cadière B, Cadière GB, Fabeck L. Evolution des douleurs  lombaires après chirurgie bariatrique. Rev Med Brux. 2015 ;36(3) :147–51. | Wrong participant population |
| K. S, M. MM, M. AK, Ramasamy C. Effectiveness of patient counseling on weight reduction in rural and urban overweight and obese patients. Int J Pharma Bio Sci [Internet]. 2011;2(3 PG-173–185):173–85. | Outcome measure not specific to LBP |
| Lavin J. Taking a load off: how losing weight can help to relieve back pain.  Talkback Mag [Internet]. 2008 Sep;22–3. | Wrong study design |
| Lee JS, Kang SJ. The effects of strength exercise and walking on lumbar function, pain level, and body composition in chronic back pain patients. J Exerc Rehabil. 2016;12(5 PG-463–470):463–70. | Not evaluating weight loss program |
| M. B, M. L, S. K, W. M, Fobi M. Resolution of knee and back pain after bariatric surgery and enhancement of functional ability: A cohort study from single center. Obes Surg [Internet]. 2019;29(5 Supplement PG-610):610. | Abstract only |
| M. D, K. D, Deitel W. The effect of weight loss on musculoskeletal pain in the morbidly obese. Obes Surg [Internet]. 2010;20(8 PG-1022–1023):1022–3. | Abstract only |
| M. FO, S. B, S. S, C. B, Kjellby-Wendt G. Increased joint pain after massive weight loss: is there an association with joint hypermobility? Surg Obes Relat Dis [Internet]. 2017 ;13(5 PG-877–881) :877–81. | Wrong study design |
| Muehlbacher M, Nickel MK, Kettler C, Tritt K, Lahmann C, Leiberich PK, et al. Topiramate in treatment of patients with chronic low back pain: A randomized, double-blind, placebo-controlled study. Clin J Pain. 2006;22(6):526–31. | Not evaluating a weight loss program |
| Orvieto R, Rand N, Lev B, Wiener M, Nehama H. Low back pain and body mass index. Mil Med [Internet]. 1994;159(1 PG-37–8):37–8. | Wrong study design |
| R. T, J.V. P, R.B. R, S. N, Balestrieri P J. Pain and obesity in the older adult. Curr Pharm Des [Internet]. 2014;20(38 PG-6037–6041):6037–41. | Wrong study design |
| Ryan CG, Vijayaraman A, Denny V, Ogier A, Ells L, Wellburn S, et al. The association between baseline persistent pain and weight change in patients attending a specialist weight management service. PloS One. 2017;12(6 PG-9):9. | Outcome measure not specific to LBP |
| S. H, Dagenais S. A supermarket approach to the evidence-informed management of chronic low back pain. Spine J [Internet]. 2008;8(1 PG-1–7):1–7. | Not evaluating weight loss program |
| Suter M, Donadini A, Romy S, Demartines N, Giusti V. Laparoscopic Roux-En-Y gastric bypass: Significant long-term weight loss, improvement of obesity-related comorbidities and quality of life. Ann Surg. 2011;254(2):267–73. | Wrong participant population |
| Szczurko O, Cooley K, Busse JW, Seely D, Bernhardt B, Guyatt GH, et al. Naturopathic care for chronic low back pain: a randomized trial. PloS One. 2007;2(9):e919. | Not evaluating weight loss program |
| S.H. L, T. F, Jordal S. No Title. Tidsskr Nor Laegeforen [Internet]. 2019;139(12 PG-). | Wrong study design |
| Vincent HK, Ben-David K, Cendan J, Vincent KR, Lamb KM, Stevenson A. Effects of bariatric surgery on joint pain: a review of emerging evidence. Surg Obes Relat Dis. 2010;6(4 PG-451–460):451–60. | Wrong study design |
| Williams A, Lee H, Kamper SJ, O’Brien KM, Wiggers J, Wolfenden L, et al. Causal mechanisms of a healthy lifestyle intervention for patients with musculoskeletal pain who are overweight or obese. Clin Rehabil [Internet]. 2019;33(6 PG-1088–1097):1088–97. | Secondary analysis of a different study |

**Appendix 3 – Report of Weight Loss and BMI Reduction**

**Appendix 3 Table.** Amount of weight loss and decrease in BMI post intervention.

| **Study** | **Baseline weight (kg) and/or BMI (kg/m^2^)** | **Post-intervention weight (kg) and/or BMI (kg/m^2^)** | **Correlation of change in weight and LBP outcomes** |
| --- | --- | --- | --- |
| Bhandari et al. 2019 | Mean BMI: 54.2 ± 8.6 kg/m^2^  Mean weight: 128.6 ± 20.8 kg | Mean BMI: not reported  Percentage of total weight loss was 22% and 31% at 6 and 12 months respectively. | Correlation between change in BMI after 1 year and NPRS for back pain was r = 0.40 (p = 0.002). |
| Hooper et al. 2007 | Mean BMI (female): 51 ± 8 kg/m^2^  BMI (1 male): 54 kg | Mean BMI (female): 36 ± 7 kg/m^2^  BMI (1 male): 39 kg  Total mean weight loss: 41 ± 15 kg | Not reported. |
| Khoueir et al. 2009 | Mean BMI: 52.25 ± 12.61 kg/m^2^  Mean weight: 144.52 ± 41.21 kg | Mean BMI: 38.32 ± 69.66 kg/m^2^  Mean weight: 105.59 ± 29.24 kg | Not reported. |
| Lidar et al. 2012 | Mean BMI: 42.8 ± 4.8 kg/m^2^  Mean weight: 119.6 ± 20.7 kg | Mean BMI: 29.7 ± 3.4 kg/m^2^  Mean weight: 82.9 ± 14.0 kg | No significant correlation was established between decrease in BMI and improvement in back pain (r = 0.231; p = 0.218). |
| McGoey et al.  1990 | Mean BMI: not reported  Mean weight: 125 kg | Mean BMI: not reported  Mean weight loss: 44 kg | States that pain relief in those that lost a moderate amount of weight (< 27kg) compared to those who lost >45 kg showed no statistical significance. Correlation analysis was not provided to allow for better interpretation. |
| Melissas et al. 2003 | Mean BMI: 48.03 ± 8.94 kg/m^2^  Mean weight: 131.9 ± 25.88 kg | Mean BMI: 33.26 ± 6.27 kg/m^2^  Mean weight: 92.3 ± 18.05 kg | Not reported. |
| Melissas et al.  2005 | Mean BMI: 47.2 ± 8.8 kg/m^2^  Mean weight: 132.5 ± 27 kg | Mean BMI: 32.9 ± 6.3 kg/m^2^  Mean weight: 92.3 ± 19 kg | Not reported. |
| Roffey et al. 2011 | Mean BMI: 44.7 ± 7.6 kg/m^2^  Mean weight: 123.0 ± 25.2 kg | Mean BMI (14 weeks): 38.2 ± 7.1 kg/m^2^  Mean weight (14 weeks): 104.3 ± 22.0 kg  Mean BMI (week 53): 39.6 ± 8.2 kg/m^2^  Mean weight (week 53): 107.9 ± 22.2 kg | % Reduction in BMI at Week 53 is positively correlated with improvement in NPRS and ODI. Correlation analysis was not provided to allow for better interpretation. |
| Silisteanu et al. 2015 | Mean weight not reported  **Control group:**  Men URBAN Mean BMI: 25.7 ± 5.2 kg/m^2^  Men RURAL Mean BMI: 29.8 ± 6.1 kg/m^2^  Women URBAN Mean BMI: 24.8 ± 4.8 kg/m^2^  Women RURAL Mean BMI: 31.1 ± 6.8 kg/m^2^  **Treatment group:**  Men URBAN Mean BMI: 25.7 ± 5.2 kg/m^2^  Men RURAL Mean BMI: 29.8 ± 6.1 kg/m^2^  Women URBAN Mean BMI: 24.8 ± 4.8 kg/m^2^  Women RURAL Mean BMI: 31.1 ± 6.8 kg/m^2^ | Mean weight not reported  **Control:**  Men URBAN Mean BMI: 25.1 ± 4.7 kg/m^2^  Men RURAL Mean BMI: 25.7 ± 5.6 kg/m^2^  Women URBAN Mean BMI: 24.8 ± 4.5 kg/m^2^  Women RURAL Mean BMI: 30.3 ± 6.4 kg/m^2^  **Treatment:**  Men URBAN Mean BMI: 22.3 ± 3.9 kg/m^2^  Men RURAL Mean BMI: 21.8 ± 65.9 kg/m^2^  Women URBAN Mean BMI: 21.1 ± 4.2 kg/m^2^  Women RURAL Mean BMI: 21.2 ± 5.2 kg/m^2^ | States that there are strong associations between BMI, VAS and QOLS following nutritional intervention, but no correlation analysis data is provided to allow for better interpretation. |
| Vincent et al. 2012 | **Control group:**  Mean BMI: 42 ± 6 kg/m^2^  Mean weight: 115 ± 22 kg  **Treatment group:**  Mean BMI: 47 ± 7 kg/m^2^  Mean weight: 125 ± 21 kg | **Control group**  Mean change in BMI: 0.5 kg/m^2^  No change in weight  **Treatment group:**  Mean change in BMI: -7.9 ± 2.5 kg/m^2^  Mean weight change: -19.4 ± 7.7 kg | Not reported. |
| Williams et al. 2018 | **Control group:**  Mean BMI: 32.1 ± 3.6 kg/m^2^  Mean weight: 90.8 ± 14.6 kg  **Treatment group:**  Mean BMI: 32.4 ± 3.5 kg/m^2^  Mean weight: 91.9 ± 16.5 kg | **Control group:**  Mean BMI (Week 6): 32.0 ± 4.1 kg/m^2^  Mean weight (Week 6): 90.2 ± 15.0 kg  Mean BMI (Week 26): 32.5 ± 4.6 kg/m^2^  Mean weight (Week 26): 93.3 ± 16.8 kg  **Treatment group:**  Mean BMI (Week 6): 32.8 ± 4.1 kg/m^2^  Mean weight (Week 6): 93.9 ± 18.0 kg  Mean BMI (Week 26): 32.7 ± 4.3 kg/m^2^  Mean weight (Week 26): 93.5 ± 17.4 kg | Not reported. |
